# Ibuprofen Removal by Graphene Oxide and Reduced Graphene Oxide Coated Polysulfone Nanofiltration Membranes

**DOI:** 10.3390/membranes12060562

**Published:** 2022-05-28

**Authors:** Asunción M. Hidalgo, María Gómez, María D. Murcia, Gerardo León, Beatriz Miguel, Israel Gago, Pilar M. Martínez

**Affiliations:** 1 Departamento de Ingeniería Química, Universidad de Murcia, Campus Universitario de Espinardo, 30100 Murcia, Spain; maria.gomez@um.es (M.G.); md.murcia@um.es (M.D.M.); pilarmaria.martizez@um.es (P.M.M.); 2 Departamento de Ingeniería Química y Ambiental, Universidad Politécnica de Cartagena, Paseo Alfonso XIII 52, 30206 Cartagena, Spain; gerardo.leon@upct.es (G.L.); beatriz.miguel@upct.es (B.M.); israel.gago@upct.es (I.G.)

**Keywords:** nanofiltration, modified membranes, graphene oxide, reduced graphene oxide, magnesium chloride, ibuprofen

## Abstract

The presence of pharmaceutical products, and their metabolites, in wastewater has become a focus of growing environmental concern. Among these pharmaceutical products, ibuprofen (IBU) is one of the most consumed non-steroidal anti-inflammatory drugs and it can enter the environment though both human and animal consumption, because it is not entirely absorbed by the body, and the pharmaceutical industry wastewater. Nanofiltration has been described as an attractive process for the treatment of wastewater containing pharmaceutical products. In this paper, the modification of a polysulfone nanofiltration membrane by coating with graphene oxide (GO) and reduced graphene oxide (RGO) has been carried out. The morphology and elemental composition of the active layer of unmodified and modified membranes were analyzed by scanning electronic microscopy (SEM) and energy-dispersive X-ray spectroscopy (EDX), respectively. Initial characterization membranes was carried out, studying their water permeability coefficient and their permeate flux and rejection coefficients, at different applied pressures, using magnesium chloride solutions. The behavior of both pristine and coated membranes against ibuprofen solutions were analyzed by studying the permeate fluxes and the rejection coefficients at different pressures and at different contaminant concentrations. The results have shown that both GO and RGO coated membranes lead to higher values of ibuprofene rejection than that of uncoated membrane, the latter being the one that presents better results in the studies of permeability, selectivity, and fouling.

## 1. Introduction

In 2019, the World Economic Forum ranked water scarcity and quality among the five most important conflicts. To deal with this situation, wastewater treatment and its subsequent reuse are used, but ordinary plants are unable to achieve the elimination of certain pollutants that are shown as emerging [1]. These contaminants are defined as chemical compounds whose monitoring is not usual, and may be artificial, natural, or anthropogenic. Although more research is required to find out their toxicity, it is certain enough that they cause adverse effects on both the environment and health [2].

The importance of the removal study of these compounds is reflected by the amount of literature in studies carried out by different researchers in relation to the risk and toxicity analysis of emerging pollutants. The study of Zhou et al. [3] is based on the development of an optimized method that examines the frequencies of concentrations above the predicted levels without effects. It is observed that the concentrations of pharmaceutical compounds were generally higher than the minimum concentration with no observable effect in the risk assessment in European surface waters. Likewise, the research of Van Gils et al. [4] is based on the development of a collection of integrated models that simulate solutions of chemical products, managing to approximate real daily mixtures. A 36% increase in the use of these drugs by 2030 is assumed.

For the removal of these compounds, there are different types of treatments that can be classified as chemical, biological, and physical. Among the chemical treatments, advanced oxidation processes can achieve the elimination of emerging contaminants, despite the complexity of their decomposition. On the other hand, the application of ultraviolet radiation, ozone, and the use of hydrogen peroxide are very common, achieving a better elimination with the use of combinations of these methods [5]. In the case of biological treatments, the use of bacteria can lead to the degradation of compounds [6]. Finally, within the physical treatments one of the main ones is the method of adsorption, divided into physiosorption and chemisorption [7]. The use of activated carbon also stands out due to its carbonaceous configuration similar to that of graphite [8]. In addition, within this type of treatments the use of membranes stands out, since they have numerous advantages, such as the need for low energy maintenance and simple operating conditions.

In fact, membranes are able to provide good results in the elimination of contaminants since they are mainly based on separation according to molecular size and pore size [9]. The study carried out by Kabbani et al. [10] is based on the consequences of the treatment with different concentrations of monovalent salt of sodium chloride of rejections of pharmaceutically active compounds using nanofiltration membranes. It is observed that the steric effect corresponds to the most efficient form of rejection in the removal of pharmaceutical substances. On the other hand, according to the research carried out by Licona et al. [11] in relation to the removal potential of pharmaceutical products by nanofiltration membranes and reverse osmosis, it is observed how the rejection depends on the morphology, hydrophobicity, porosity, charge, and the molecular cutting value of the membrane, in addition to the molecular size, load, hydrophobicity, and molecular weight of the contaminants and feed water.

Regarding the study carried out by Heo et al. [12] it is observed how ultrafiltration membranes provide a better elimination of emerging organic contaminants consisting of lower polarity, greater hydrophobicity, and greater volatility, thus deducing that hydrophobic adsorption predominates in the elimination through these membranes. Therefore, they can then be employed as pretreatment with the subsequent use of direct and reverse osmosis membranes.

Likewise, in the research carried out by Shad [13], it is deduced how the treatment with microfiltration membranes provides good results in the elimination of organic matter in suspension, although it does not achieve the elimination of inorganic compounds in solution. Consequently, these membranes must be applied as a pretreatment and the application of reverse osmosis membranes must be improved.

Within this field, an important research advance is the use of modified membranes that present greater selectivity and better yields. The main advantages are based on the increase in chemical resistance and service life, the improvement of separation and rejection and the decrease in fouling. Membranes modified by graphene, graphene oxide, and reduced graphene oxide are known to provide better results in numerous applications compared to traditional membranes. For this reason, the amount of research has increased in recent years [14,15,16,17,18].

In the research carried out by Fathizadeh et al. [15] on the printing of graphene oxide on ultrafine nanofiltration membranes, it was found that membranes printed with graphene oxide have a higher permeability to water and also provide better rejections of small organic molecules. In addition, the modified membranes demonstrated long-term stability and optimal nanofiltration performance in relation to the removal of pharmaceutical contaminants in the water.

In the light of recent studies, the main objective of this work has been to carry out the modification of a nanofiltration membrane by using graphene oxide and reduced graphene oxide for its application in the removal of emerging contaminants such as, in this case, ibuprofen.

Ibuprofen has been selected as target specie in this study because it is one of the most consumed non-steroidal anti-inflammatory drugs, with a global production of 15,000 tons/year [19]. It is mainly prescribed for the treatment of rheumatoid arthritis and osteoarthritis, although it can also be applied for the alleviation of mild to moderate pain, inflammation and fever [20]. Moreover, it can enter the environment though both human and animal consumption, because it is not entirely absorbed by the body, and through pharmaceutical industry wastewater. As a result, ibuprofen concentrations of up to 1.9 μg/L and 25 ng/L have been found in surface and drinking water, respectively [21,22].

## 2. Materials and Methods

### 2.1. Materials

#### 2.1.1. Reagents

Ibuprofen (≥98%), C_13_H_18_O_2_, molecular weight 206.28 g/mol, supplied by Sigma-Aldrich (Barcelona, Spain); ethanol, C_2_H_6_O, molecular weight 46.07 g/mol, supplied by Panreac (Castelar del Vallés, Spain); magnesium chloride hexahydrate, MgCl_2_·6H_2_O (for analysis grade), molecular weight 203.30 g/mol, supplied by Panreac (Castelar del Vallés, Spain); and graphene oxide (99%) and reduced graphene oxide (80% C) were obtained from Abalonyx (Oslo, Norway).

#### 2.1.2. Membrane (Alfa Laval-NF)

The polysulfone nanofiltration membrane used in this study was supplied by Alfa Laval (Madrid, Spain). Its main technical characteristics are shown in Table 1.

### 2.2. Methods

The procedures performed are as follows:

#### 2.2.1. Membranes Modification

Dispersions of graphene oxide (GO) and reduced graphene oxide (RGO), at a concentration of 0.15% *w*/*v* were prepared by dispersing GO or RGO in distilled water by sonication, using a Branson 450D sonicator (Emerson Ultrasonic Corporation, Madrid, Spain), by application of 2 cycles of amplitude of 30% of 5 min with pulses of 5 s on and 5 s off. Next, the dispersions were vacuum filtered using a Büchner funnel where the membrane had previously been placed. The so prepared GO or RGO coated membranes were left to dry for 24 h at room temperature.

#### 2.2.2. Morphological Characterization of the Membranes

The active layers of native and modified membranes were analyzed, before and after finishing the experiments, by scanning electron microscopy (SEM) and energy-dispersive X-ray spectroscopy (EDX), using a SEM HITACHI S-3500N apparatus (Hitachi High-Technologies Corporation, Tokyo, Japan), equipped with an EDX XFlash 5010 analysis system (Brukers AXS, Karlsruhe, Germany) [23].

#### 2.2.3. Physico-Chemical Characterization of the Membranes

Experimental tests were performed in an INDEVEN flat membrane test module (Bilbao, Spain) that allows to obtain data concerning the behavior of the membranes in cross flow conditions with a reduced surface area, low feed, and short times [23]. Experiments were carried out by recycling of both concentrate and permeate in order to keep the feed concentration practically constant and so simulate a continuous process in a quasi-stationary state. In each experiment, the steady state was allowed to be reached by operating the module for 30 min and, thereafter, two samples were taken from each of the feed and permeate streams, with a time interval of 5 min. The membrane effective area was 0.003 m^2^.

Water permeability tests were carried out with pure water as feed using pressures of 10, 15 and 20 bars and a constant flow rate of 150 L/h. Water permeability coefficient (*A_w_*) was obtained by the equation:

(1) $$J_{w}=A_{w}\;\cdot \;\left(\Delta P-\Delta \Pi \right)$$

where *J_w_* is the solvent permeate flux (kg/m^2^ s), *A_w_* is the solvent permeability coefficient (s/m) and Δ*P* and Δ*Π*, are operating and osmotic pressure, respectively (Pa). *A_w_* can be determined as the slope of the representation of *J_w_* versus ΔP.

In the study of permeate fluxes and rejections of magnesium chloride and of ibuprofen, 1 g/L feed magnesium chloride solutions, and 5, 7.5, and 10 ppm feed ibuprofen solutions (at neutral pH) were used, respectively, both at pressures of 10, 15, and 20 bars and at constant flow rate of 150 L/h. Permeate fluxes (*J_p_*) and rejections (*r*) were determined by the following equations:

(2) $$J_{p}=\frac{Q_{p}}{S}$$

(3) $$r=\frac{\left(C_{f}-C_{p}\right)}{C_{f}}$$

where *J_p_* (kg/(m^2^·s) is the permeate flux, *Q_p_* is the mass flow rate (kg/s) and *S* is the membrane active area (m^2^), *r* is the rejection coefficient, and *C_f_* and *C_p_* are the solute concentration in the feed and permeate stream, respectively (ppm).

#### 2.2.4. Analytical Methods

The concentrations of magnesium chloride and of ibuprofen in both the feed and in the permeate streams were determined by measuring, respectively, electrical conductivity by an EC-Metro GLP 31 conductivity meter (Crison, Hospitalet de Llobregat, Spain), and ultraviolet light absorption, at 195 nm, by a Helios Alpha spectrophotometer (Thermo Fischer Scientific, Madrid, Spain), using previously obtained calibration curves.

## 3. Results and Discussion

### 3.1. Morphological Characterization of the Membranes

In order to know if the modifications made have been effective at microscopic levels, the following morphological study is carried out using SEM scanning electron microscopy.

**Figure 1:**
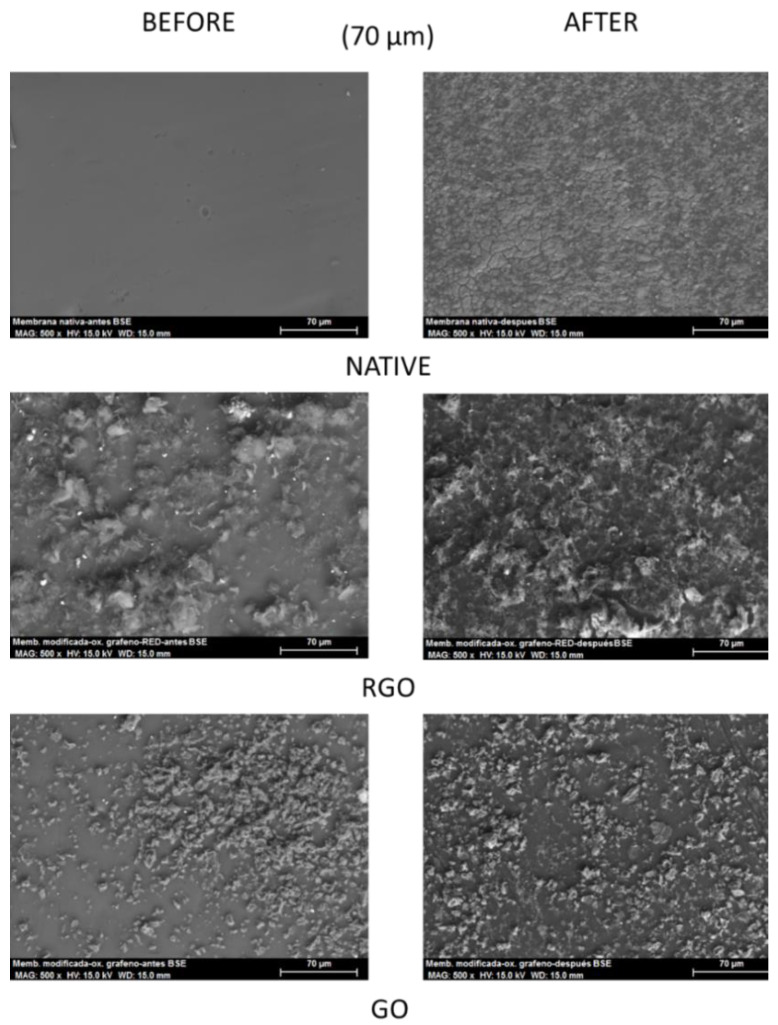
SEM images of the membranes (native and modified).

Figure 1 shows that the coating of the polysulfone membrane with GO and RGO results in an increment of its superficial roughness due to the interfacial enrichment of nanomaterials onto the polysulfone membrane. This increment is more significant in the RGO coated membrane than in the GO coated membrane.

Additionally, it can be noticed how, after the assays with the three membranes, some substances appear that dirty the active layers, causing detachments and alterations in the modified membranes.

Similar relation between native and cellulose acetate butyrate membrane modified with graphene-based nanomaterials has been described by other authors [24].

**Figure 2:**
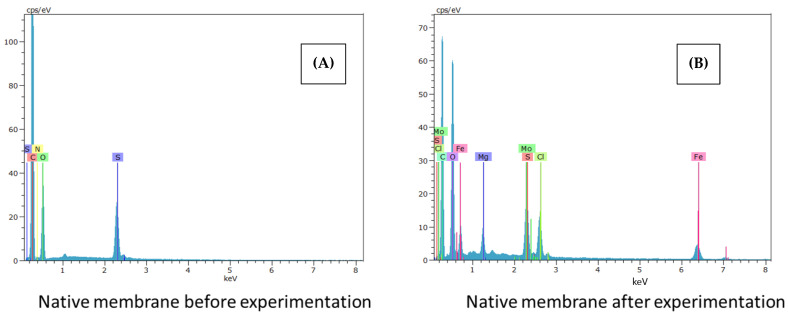
SEM-EDX spectra obtained with (**A**) the native membrane before and (**B**) after the experiments.

**Figure 3:**
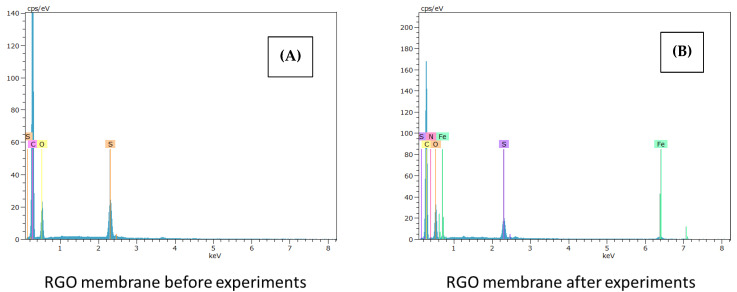
SEM-EDX spectra corresponding to the membrane modified with reduced graphene oxide (**A**) before and (**B**) after the experiments.

**Figure 4:**
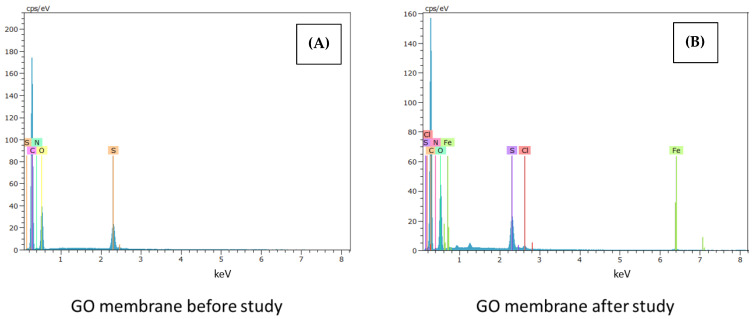
SEM-EDX spectra corresponding to the membrane modified with graphene oxide (**A**) before and (**B**) after the experiments.

The following Figure 2, Figure 3 and Figure 4 show the SEM-EDX spectra corresponding to the native membrane, the membrane modified with reduced graphene oxide and, finally, the membrane modified with graphene oxide, respectively. These analyses have been carried out both at the beginning and at the end of the experiments.

For all the membranes, more elements can be seen in the spectra after carrying out the experiments, due to the interactions of the active layer with ibuprofen. Furthermore, in the case of the spectra of the membrane modified with graphene oxide, more peaks are shown both at the beginning and at the end of the tests compared to the membrane modified with reduced graphene oxide, thus assimilating it to the behavior of the native membrane.

According to the literature, ibuprofen has been documented to attach to chromium-based metal frameworks and it is noted that the experimental test equipment is comprised mainly of components that consist of stainless steel (16% chromium, 10% nickel, 2% molybdenum, and less than 0.02% carbon) [25].

Therefore, the presence of Fe, Mo and other elements in the SEM-EDX spectra for native, GO, and RGO modified membranes after experimentation may be due to the accessories of the experimental equipment used to carry out the tests.

The materials of the pump, valves, and feed tank, are made of stainless steel with metallic alloys, and can justify the presence of these elements in the membranes after carrying out the tests.

In addition, in the spectra obtained at the end of the tests with the modified membranes, the presence of a greater number of peaks at the beginning of the graphs is observed. Therefore, the modifications are competent after the treatment of the contaminant.

### 3.2. Physico-Chemical Characterization of the Membranes

#### 3.2.1. Solvent Permeability

Table 2 shows the solvent permeability coefficients obtained for the three membranes. When comparing the results obtained with the research of Tahaikt et al. [26], both the native membrane and the one modified with reduced graphene oxide have an order of magnitude close to the permeability coefficient of 1.225 × 10^−6^ m^3^/m^2^‧s obtained with the NF90 membrane of the cited work. The important decrease of water permeability coefficient in the GO coated membrane with respect to the pristine one can be explained by the interactions between the hydrogen of the carboxyl and hydroxyl groups present in GO, and the oxygen of the sulfone groups of polysulfone membrane, which leads to the interfacial enrichment of GO onto the polysulfone membrane by a self-assembly process. This interaction results in smaller membrane pore sizes caused by the stacked positioning of GO nanosheets on the membranes, resulting in dense GO nanochannels with great enrichment of interfacial GO onto the membrane surfaces [27]. The number of carboxyl and hydroxyl groups is much lower in the RGO modified membrane, therefore, this effect, and consequently the decrease in the permeability of water with respect to the original membrane, is also much lower.

#### 3.2.2. Membranes Selectivity

**Figure 5:**
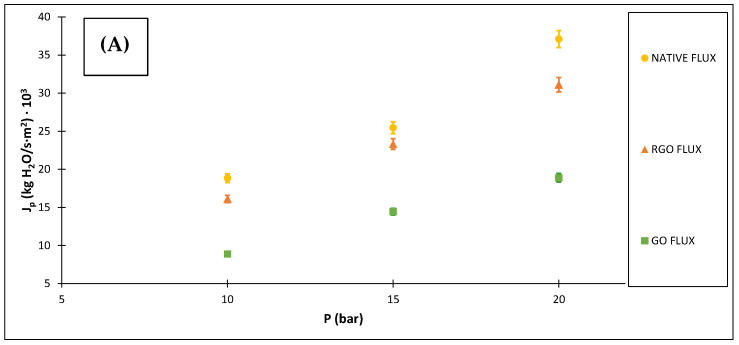

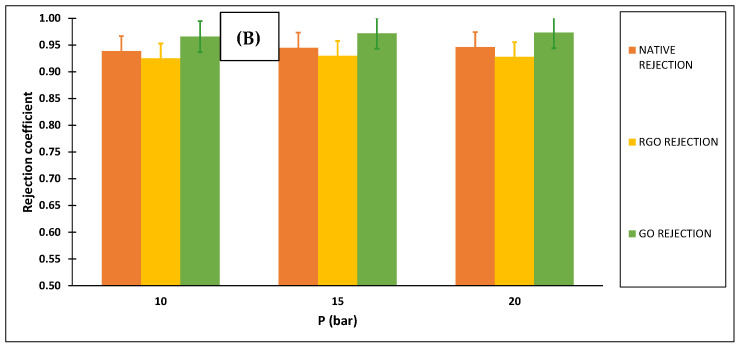
Permeate mass flows (**A**) and rejections of saline solutions (**B**) against pressures.

Figure 5 shows both the permeate flows and the rejection coefficients achieved with the native membrane and the modified ones in the assays with the saline solutions. It can be seen that when the operating pressure increases, there is an increase in the permeate flow, because an increase in hydraulic pressure achieves a greater flow. Regarding the rejection coefficient, it is observed that for both the native membrane and the modified ones this value is practically the same for the pressures tested, obtaining a higher selectivity with the application of the membrane modified with graphene oxide.

The results can be compared with the study of Peng et al. [28] on nanofiltration membranes of piperazine-trimesoyl chloride polyamide and the modification made by a strong electrolyte monomer containing a multiple of amines and quaternary ammonium. The experimentation of the selectivity is carried out with a solution of 1 g/L of MgCl_2_ with a pressure of 6 bars and a temperature of 25 °C and a permeate flow close to 36 L/m^2^‧h is achieved, reaching a rejection close to 0.47 before the modification. After the modification of the active layer, a permeate flow value of approximately 109 L/m^2^‧h and a rejection close to 0.46 are obtained. So, the rejections obtained in the present research are higher, while the permeate flows reached with the modified piperazine-trimesoyl chloride polyamide membrane are similar to those achieved for the 20-bar pressure with the native membranes and the ones modified with reduced graphene oxide. Additionally, the permeate flow achieved with the membrane before the alteration with the strong electrolyte monomer is very close to that obtained in this research with the membrane modified by graphene oxide for the pressure of 10 bars.

The research of Park et al. [29] focuses on the modification by inkjet printing process of a flat sheet nanofiltration membrane, composed of thin film where single-walled carbon nanotubes were placed. Saline permeate flow results of 19 L/m^2^‧h‧bar and a rejection coefficient close to 0.55 are obtained under conditions of 1 g/L of MgCl_2_ dissolution and with a pressure of 4 bars. Therefore, the values achieved in this research are higher for both parameters.

#### 3.2.3. Ibuprofen Removal

In order to know the removal efficiency of the contaminant using the different membranes, the influence of the following variables has been studied: operating pressure and ibuprofen feed concentration.

**Figure 6:**
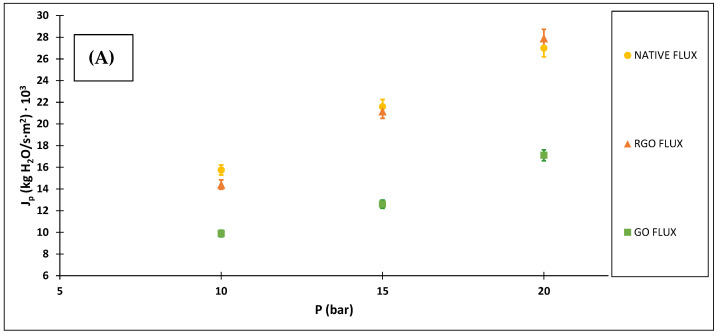

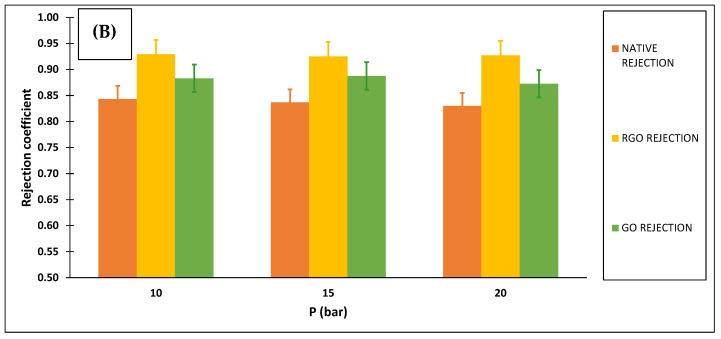
Permeate mass flows (**A**) and rejection coefficients (**B**) using 10 ppm ibuprofen solutions against different operating pressures.

Figure 6 shows the values of the permeate mass flows and rejections against the three pressures studied, using ibuprofen solutions of 10 ppm for the three membranes.

As it can be seen in Figure 6, the permeate mass flow increases with the operating pressure. This almost linear increase indicates that the fouling effect and the polarization effects are not very significant. However, the values of the rejection coefficients for each membrane remain practically constant within the range of pressures studied.

It can be seen how the modified membranes provide higher rejection coefficients compared to the native membrane, achieving better results with the membrane modified with reduced graphene oxide. On the opposite, the native membrane provides a higher mass flow, followed by the membrane modified with reduced graphene oxide and placing the membrane modified with graphene oxide in the last place.

The solvent permeability values of the GO membrane were lower than the ones of the RGO and native membrane, this reduction upon high loadings of nanomaterial has been previously reported in several studied [30,31] and can be attributed to the presence of a tipping mass percentage of nanofiller [32,33].

The rejection coefficients of the RGO and GO membranes were higher than that of the native membrane. The negative charge of both, modified membranes and ibuprofen, at neutral experimental pH, leads to an increase in the repulsion of modified membranes and ibuprofen. [17,18,27]. The higher rejection of the RGO coated membrane, despite its lower number of negatively charged groups at the working pH, may be related to additional interactions [17] between the hydrophobic zones of both ibuprofen molecule and RGO modified membrane.

**Figure 7:**
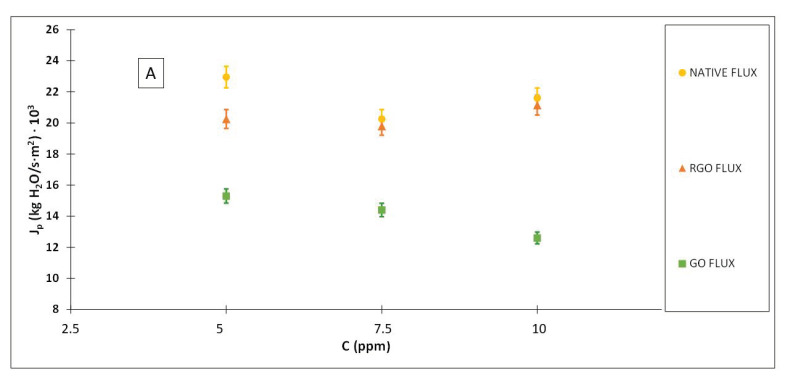

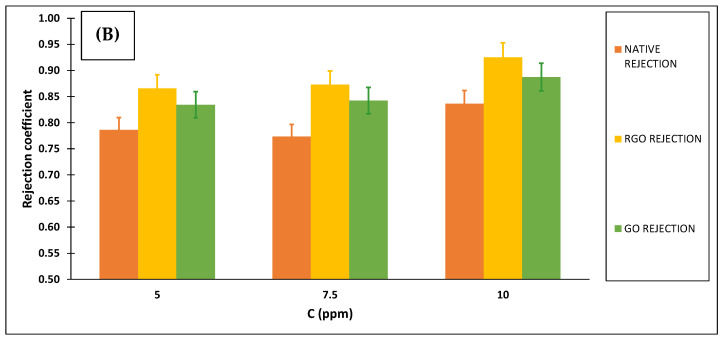
Permeate mass flows (**A**) and rejection coefficients (**B**) obtained at a pressure of 15 bar using ibuprofen solutions of different concentrations.

Figure 7 shows the results obtained for the rejection coefficients and the permeate mass flows when using different concentrations of ibuprofen with a pressure of 15 bars.

It is observed how the highest rejection coefficients achieved correspond to the modified membranes, highlighting the membrane modified with reduced graphene oxide. Again, a greater flow is achieved with the native membrane, and the lowest ones with the membrane modified with graphene oxide.

Although other authors have worked at different pH values, in this work the experiments were carried out under conditions of neutral pH due to the isoelectric properties of the contaminant and the membranes used. However, values of permeate flows and rejection coefficients similar to those obtained in this study can be found [34,35,36].

In the research carried out by Bareera et al. [37] on the behavior of paracetamol, diclofenac, and ibuprofen with thin-layer nanofiltration membranes, normally used on treatments with organic compounds of great molecular weight, an ibuprofen rejection coefficient close to 0.81 at neutral pH using an NF50 membrane is achieved. Therefore, the rejection coefficients obtained in our research are higher, except for those corresponding to the native membrane with the concentrations of 5 and 7.5 ppm for the pressure of 15 bars.

A comprehensive comparison of membrane separation performance with other reported/published data is shown in Table 3.

Even though several studies have been carried out using nanofiltration polymeric membranes for the removal of ibuprofen, very few of them have employed modified membranes with the aim of obtaining greater permeate fluxes and performance. As a result, the development of further studies on this topic would be very interesting.

#### 3.2.4. Fouling Study and Membrane Deterioration

In this study, a comparison of the initial parameters with the final ones is carried out to determine the membranes fouling.

Table 4, Table 5 and Table 6 show the initial and final mass flow values from both the permeability and selectivity studies, in addition to the rejection coefficients of the saline solutions and the fouling factor (F) of the native membrane, the membrane modified with reduced graphene oxide, and, finally, the membrane modified with graphene oxide, respectively.

In Table 4 it can be seen how the values of permeate mass flow decrease after carrying out the experiments both in the permeability and selectivity tests. On the other hand, the fouling factor is reduced by increasing pressure. In addition, it is observed how the rejection capacity increases after the tests, which is a characteristic behavior of the membrane aging.

Table 5 shows how fouling does not affect the test carried out with the pressure of 10 bars. On the contrary, the fouling factor corresponding to the pressure of 20 bar presents a considerable increase in relation to the one obtained at 15 bars. A decrease in permeate flow is observed in almost all tests. In addition, the final rejection decreases, this could be due to the compound detaching from the active layer.

From Table 6 it can be observed that the permeate flow values of the permeability study for pressures of 15 and 20 bar increase after the tests and there is no significant fouling effect. The rejection coefficients of the saline solutions decrease with respect to the initial ones, although the final permeate flows are very similar.

The decrease of the rejection coefficients of the modified membranes is verified by the morphological study, where the deterioration of these membranes after the experiments is appreciated and can cause the compound detaching from the active layers (Figure 1).

Finally, the presence of a higher fouling, the high rejection coefficients of the final selectivity assays, in addition to the excellent results of the rejection coefficients obtained in the ibuprofen removal with the membrane modified with reduced graphene oxide compared to the membrane modified with graphene oxide can be justified with the SEM-EDX spectra located in Figure 2, Figure 3 and Figure 4, where a behavior of the membrane modified with reduced graphene oxide less similar to the native membrane is observed in contrast to the membrane modified by graphene oxide.

As for other fouling studies, the one carried out by Marszałek et al. [47] is based on the treatment of a nanofiltration membrane exposed to photooxidation in order to achieve a reduction in fouling. The fouling factor values for reversible and irreversible aging are approximately 0. 48 and 0. 69, respectively. Compared to the results obtained there is a significant difference, being the highest factors in the present research those achieved with the native membrane.

## 4. Conclusions

In this paper, the modification of a polysulfone nanofiltration membrane by coating with GO and RGO has been carried out. The coating of the polysulfone membrane with GO and RGO results in an increment of its superficial roughness. Water permeability coefficient of the modified membranes is lower than that of native membrane, as a result of the interactions between the hydrogen from the carboxyl and hydroxyl groups present in GO and RGO, and the oxygen of the sulfone groups present in the polysulfone membrane. These interactions lead to the interfacial enrichment of the nanomaterials coating the polysulfone membrane by a self-assembly process, which results in smaller membrane pore sizes. RGO and GO coated membranes show higher ibuprofen rejection coefficients than the native membrane. These results could be explained due to the negative charge present in RGO and GO coated membranes which, as a result of the negative charge of ibuprofen, leads to an increase in the repulsion of modified membranes and ibuprofen, at neutral pH. The higher ibuprofen rejection coefficient obtained for the RGO coated membrane, despite its lower number of negatively charged groups at the working pH, may be related to additional interactions between the hydrophobic zones of both ibuprofen molecule and RGO modified membrane.

## Figures and Tables

**Table 1:** Main technical characteristics of nanofiltration membrane.

Manufacturer	Alfa Laval (Denmark)
Product denomination	NF
Composition	Polysulfone
Pore size (Da)	300
Maximum pressure (N m^−2^)	55 × 10^5^
Operating pressure range (N m^−2^)	15–42 × 10^5^
Temperature range (°C)	5–50
Cl free concentration (ppm)	<0.1
pH range (T_reference_ = 25 °C)	3–10

**Table 2:** Solvent permeability coefficients.

Coefficient of Permeability to Solvent 10^−8^ (s/m)		
Native	RGO	GO
17.15	16.11	8.33

**Table 3:** Comparison of membrane performance in the removal of ibuprofen.

Membrane	Material	Experimental Conditions	Permeate Flux (L/m^2^h)	Rejection (%)	Reference
NF	Aromatic polyamide	pH = 7.5 P = 445–504 kPa	-	45	(Yoon et al., 2007) [38]
NF 4040	Polypiperazine-amid thin-film composite	pH = 6.3 C = 250 ng/L P = 60–70 psi	20.4	100	(Bellona et al., 2007) [39]
NF90 NF270 TFC-SR2	Polyamide thin-film with a microporous polysulfone	pH = 4–9.8 pH = 6.3–9.8 pH = 9.8	-	100 99 84	(Nghiem et al., 2007) [40]
TS80 DESAL HL	Cross-linked aromatic polyamide top layer	pH = 6.5–7.5 C = 2 µg/L P = 5 bar	-	99 98	(Verliefde et al., 2009) [41]
NF270 NF90	Thin aromatic or semiaromatic polyamide	pH = 7.4–7.6 C = 2 µg/L P = 12 bar	41.0	99 99	(Alturki et al., 2010) [42]
NF200 NF90	Aromatic polyamide	pH = 6–7 C =2 µg/L P = 12 bar	-	89 96	(Yangali Quintanilla et al., 2010) [43]
MPS-34 TFC-SR2 NF270	Polysulfone composite Polysulfone composite Polyamide thin-film composite	pH = 8 P = 5 bar	-	99 58 95	(García-Ivars et al., 2017) [44]
Ceramic membrane Ceramic GO	Ceramic membrane Ceramic GO	pH = 7 C = 10 µM P = 3 bar	25.1 14.4	70 92	(Chu et al., 2017) [27]
NF50 NF10	Sulfonated polyethersulfone	pH = 6–7	-	80.54 12	(Bareera et al., 2020) [37]
NF270 TS40	Polyamide thin-film composite Polypiperazine amide	pH = 4 C = 400 µg/L	42.4	20–30 37–42	(Higgins and Duranceau, 2020) [45]
G1 G2 G3	Polymer inclusion membrane G1 = 0.15% GO G2 = 0.45% GO G3 = 0.75% GO	pH = 2 C = 10 mg/L P = 100 psi	-	70 75 77	(Ahmad et al., 2021) [36]
AFC30 AFC40 AFC80	Polyamide	pH = 7 C = 1 mg/L P = 2 MPa	34.2	98 98 90	(Kudlek et al., 2015) [46]
NF270	Polyamide thin-film composite	pH = 7 C = 10 mg/L P = 130 psi	-	85	(Kabbani et al., 2021) [10]
NF GO RGO	Polysulfone GO 0.15% RGO 0.15%	pH = 7 C = 7.5 mg/L P = 15 bar	75.6 52.2 72.0	77 85 88	This work

**Table 4:** Initial and final permeate mass flows from the permeability and selectivity tests, rejection results of the saline solutions, and fouling factor of the native membrane.

Native Membrane				
P (bar)	Permeability			
	Initial (J_w_ (kg H_2_O/s m^2^)10^3^)	Final (J_w_ (kg H_2_O/s m^2^)10^3^)	F	
10	19.156	16.111	0.159	
15	28.053	24.444	0.129	
20	36.304	32.222	0.112	
**P (bar)**	**Selectivity**			
	**Initial**		**Final**	
	**J_p_ (kg H_2_O/s m^2^)10^3^**	**Rejection coefficient**	**J_p_ (kg H_2_O/s m^2^)10^3^**	**Rejection coefficient**
10	18.826	0.939	16.111	0.976
15	25.471	0.945	24.444	0.978
20	37.099	0.946	31.667	0.976

**Table 5:** Initial and final permeate mass flows from the permeability and selectivity tests, rejection results of the saline solutions, and fouling factor of the membrane modified with reduced graphene oxide.

RGO Membrane				
P (bar)	Permeability			
	Initial (J_w_ (kg H_2_O/s m^2^)10^3^)	Final (J_w_ (kg H_2_O/s m^2^)10^3^)	F	
10	15.556	16.111	−0.036	
15	24.444	23.333	0.045	
20	31.667	28.889	0.088	
**P (bar)**	**Selectivity**			
	**Initial**		**Final**	
	**J_p_ (kg H_2_O/s m^2^)10^3^**	**Rejection coefficient**	**J_p_ (kg H_2_O/s m^2^)10^3^**	**Rejection coefficient**
10	16.111	0.925	14.444	0.846
15	23.333	0.930	23.889	0.825
20	31.111	0.928	30.000	0.835

**Table 6:** Initial and final permeate mass flows from the permeability and selectivity tests, rejection results of the saline solutions, and fouling factor of the membrane modified with graphene oxide.

GO Membrane				
P (bar)	Permeability			
	Initial (J_w_ (kg H_2_O/s m^2^)10^3^)	Final (J_w_ (kg H_2_O/s m^2^)10^3^)	F	
10	11.667	11.111	0.048	
15	15.000	17.222	−0.148	
20	20.000	22.778	−0.139	
**P (bar)**	**Selectivity**			
	**Initial**		**Final**	
	**J_p_ (kg H_2_O/s m^2^)10^3^**	**Rejection coefficient**	**J_p_ (kg H_2_O/s m^2^)10^3^**	**Rejection coefficient**
10	8.889	0.966	8.333	0.790
15	14.444	0.972	13.333	0.831
20	18.889	0.973	18.333	0.858

## Data Availability

The data presented in this study are available on request from the corresponding author.
